# Uncertain tourist expressions and data-driven perceived destination image construction: a probabilistic linguistic and multi-criteria decision framework

**DOI:** 10.1038/s41598-026-53342-5

**Published:** 2026-05-20

**Authors:** Yong Qin, Xiaolin Zhao, Luwei Wang

**Affiliations:** 1 College of Management Science, Chengdu University of Technology, Chengdu, 610059 People’s Republic of China; 2 School of Hotel and Tourism Management, The Hong Kong Polytechnic University, 17 Science Museum Road 818, TST East, Kowloon, Hong Kong SAR People’s Republic of China; 3 School of Digital Economy and Management, Sichuan Technology and Business University, Meishan, 620036 People’s Republic of China

**Keywords:** Probabilistic linguistic modeling, Uncertain tourist expressions, Destination image analytics, Topic–sentiment integration, Multi-criteria decision making, Information systems and information technology, Mathematics and computing

## Abstract

Understanding how tourists mentally construct destination images from online information has become increasingly important, yet the textual expressions produced by tourists in online reviews often contain vague, uncertain, and multi-interpretable meanings. This study develops an integrated analytical framework to capture and utilize such ambiguity for destination image construction and recommendation. First, probabilistic linguistic term sets (PLTSs) are employed to formally model the uncertain and fuzzy components embedded in tourists’ online expressions, enabling a more fine-grained representation of perceived image attributes. Then, latent Dirichlet allocation (LDA) and sentiment analysis are applied to large-scale online reviews to identify thematic structures and emotional orientations underlying tourist discourse. By synthesizing probabilistic linguistic evaluations with extracted topics and sentiments, a multidimensional destination image is constructed that reflects both the semantic diversity and evaluative uncertainty of user-generated content. To transform these insights into decision support, the study further integrates PL-entropy weighting with the PL-MACBETH multi-criteria decision-making approach to generate rational and explainable destination recommendations. A real-world case study verifies the model’s effectiveness, demonstrating that the PLTS-based representation significantly improves the interpretation of perceived relevance and importance, and the proposed recommendation mechanism yields consistent and robust decision outcomes. The findings contribute to both tourism research and practice by offering a methodological pathway to better leverage ambiguous online expressions in destination evaluation and recommendation.

## Introduction

In the context of the thriving tourism industry, competition among tourist destinations has intensified, while the outbreak and spread of the COVID-19 pandemic have further brought unprecedented challenges to this competitive landscape^1^. The impact of the pandemic is not limited to economic aspects but has also profoundly influenced destination image formation and development. Destination image, which affects tourists’ willingness to visit and destination competitiveness, has therefore become a key concern for researchers and practitioners^2^. Destination image refers to tourists’ overall perception and impression of a destination, covering multiple dimensions such as environment, services, infrastructure, and transportation^3^. It is generally categorized into projected image and perceived image. The projected image is constructed through destination-led promotion and marketing activities, but may deviate from reality due to selective or exaggerated information, thereby affecting tourists’ actual experience^4,5^. In contrast, the perceived image is formed based on tourists’ collected information and on-site experiences, making it more authentic and informative^6^. With the deep integration of the tourism industry and the Internet, online textual expressions generated by tourists after their travel have become an important and increasingly reliable source for capturing perceived destination image, providing new opportunities for related research^7^.

The tourism industry relies heavily on information, as travelers base their decisions on gathering and comparing destination-related information^8^. Quality information services play a crucial role in guiding tourists’ destination choices. In the digital age, social media and Internet have provided travelers with ample opportunities to access and share travel-related information and experiences. This has led to a proliferation of online reviews and comments about tourist destinations. Online reviews have become a primary means for travelers to express their travel preferences and reflect their perceptions of destination image^9^. Prospective travelers increasingly rely on peer evaluations from online platforms to assess the merits and drawbacks of tourist destinations. However, as the tourism industry expands, the volume of data and information grows exponentially. Multiple websites and platforms accumulate various layers of information, resulting in information overload. Consequently, potential tourists face challenges in navigating this vast information landscape, leading to difficulties in making informed travel decisions. To address this challenge, the application of recommendation algorithms has emerged as one of the most effective solutions^10,11^. In particular, research on destination recommendation strategies that combine tourists’ online expressions with natural language processing techniques has gained recognition among researchers. Simultaneously, considering various dimensions when assessing the performance of different entities is often the cornerstone of recommendation algorithms. Consequently, issues related to tourist destinations and their recommendations are often abstracted into a multi-criteria decision making (MCDM) problem^4,5^. In recent years, tourist destination selection and recommendation have attracted growing scholarly attention. Existing studies have employed a variety of recommendation approaches, including collaborative filtering, content-based recommendation, knowledge-based recommendation, and hybrid recommendation models, to support travelers’ destination decision-making. In parallel, MCDM methods have been increasingly introduced into tourism research to evaluate destinations across multiple dimensions such as service quality, transportation accessibility, tourist experience, cultural value, and attraction appeal^12^. Although these methods have demonstrated practical value in destination ranking and recommendation, most existing approaches rely on deterministic ratings, explicit preference information, or coarse-grained sentiment representations. Limited attention has been given to the ambiguity and uncertainty embedded in tourists’ online expressions, which may reduce the interpretability and robustness of recommendation outcomes^13^.

However, in recent years, numerous scholars both domestically and internationally have recognized the presence of a certain degree of fuzziness and uncertainty in user-generated online text expressions^14^. In such scenarios, traditional decision models may struggle to effectively address complex situations and lack adaptability to uncertainty^15^. In contrast, fuzzy theory offers a more flexible and efficient perspective for handling such uncertainties^16^. Through fuzzy theory, we can incorporate fuzziness and uncertainty into the decision-making process, thereby better adapting to real-world circumstances and providing travelers with recommendations that better align with their actual needs^17,18^. Thus, various derivative fuzzy theories have been ingeniously applied in this field, including intuitionistic fuzzy set, picture fuzzy set, and type-2 fuzzy set. It is evident that these earlier theories and methods capture the subjectivity and uncertainty in tourists’ online expressions and, in conjunction with MCDM principles, have paved the way for effective recommendation strategy research for different types of tourists^19^. More recently, probabilistic linguistic term set (PLTS) is demonstrated to efficiently and reasonably capture the fuzziness and complex uncertainty within tourists’ online expressive information^20^. This is because PLTS serves as an effective tool for addressing information ambiguity and distinguishing the importance of various terms. Compared with other fuzzy modeling approaches such as intuitionistic fuzzy sets, picture fuzzy sets, and type-2 fuzzy sets, PLTS offers several advantages in modeling tourists’ online expressions. Firstly, PLTS allows multiple possible linguistic evaluations to be simultaneously represented together with their associated probability distributions, which better reflects the ambiguity, hesitation, and distributional diversity commonly observed in user-generated reviews. Secondly, unlike intuitionistic or picture fuzzy approaches that mainly focus on membership structures, PLTS preserves richer linguistic semantics while explicitly quantifying the relative likelihood of alternative expressions. This is particularly suitable for tourism review data, where users often express mixed or uncertain opinions rather than a single deterministic sentiment. Therefore, PLTS provides a more flexible and fine-grained mechanism for representing uncertain tourist perceptions in this study. For instance, Luo et al.^21^ based on online evaluations of China’s 5A-level tourist destinations, developed a comprehensive multidimensional evaluation index system. They applied PLTS and the IDOCRIW-COCOSO model to address the issue of tourist destination selection.

A review of existing research reveals that tourists’ online expressive information has been widely used in constructing the perceived destination image. However, a research gap persists in the literature. Although online review data have been widely used in prior studies to analyze tourists’ sentiments, satisfaction, and destination perceptions, most existing studies primarily rely on sentiment polarity, rating aggregation, or deterministic text representations. Limited attention has been paid to the ambiguity, hesitation, and uncertainty embedded in tourists’ expressive information itself. Therefore, the novelty of this study does not lie in adopting tourists’ online reviews as a data source, but in explicitly modeling the uncertain and fuzzy characteristics of tourist expressions for destination image construction and recommendation. In the previous research on tourists’ online expressive information, most studies have centered on online review data to investigate tourists’ emotional inclinations, satisfaction, and subsequently construct the perceived destination image. On the one hand, this approach tends to have an overly coarse granularity, which can lead to the omission of a substantial amount of valuable information. On the other hand, when utilizing tourists’ expressive information to construct the perceived destination image, many studies have overlooked the inherent fuzziness and uncertainty, failing to effectively characterize and represent them.

To bridge the gaps identified in the existing literature, the objective of this study is to investigate the construction of destination image driven by fuzzy expression information from travelers and to explore recommendation algorithm based on MCDM. Specifically, this study initially employs the LDA topic extraction model to identify the main perceptual dimensions within tourists’ online review data. Subsequently, it utilizes sentiment analysis techniques to extract PLTS information for different perceptual dimensions of tourist destinations, achieving the construction of the perceived destination image driven by PLTS information. Finally, this study applies the proposed probabilistic linguistic MACBETH (PL-MACBETH) MCDM method to the problem of ranking tourist destinations, thus completing the recommendation order for various tourist destinations and providing references for travelers’ decision-making. The research findings unequivocally demonstrate the efficacy of PLTS in elucidating destination perception. Through comparative analysis, the validity and effectiveness of the proposed recommendation method have been confirmed. To ensure conceptual clarity and consistency, this study adopts “destination image” as the unified terminology throughout the manuscript. Specifically, “perceived destination image” is used to refer to tourists’ subjective and experience-based evaluations derived from online reviews, whereas “destination image” is used as the general construct encompassing both cognitive and affective dimensions. All other variant expressions are standardized accordingly.

The remainder of this study is organized as follows. “Preliminaries” section specifies some basic knowledge of PLTS and the traditional MACBETH method. “Methodology” section describes the methodology applied in this study. “Case study” section gives a case study. “Discussions” section presents some discussions. Finally, “Conclusions” section draws conclusions.

## Preliminaries

### Probabilistic linguistic term set

In real-world decision-making, when experts qualitatively express their preferences for evaluating things, they often consider several possible linguistic terms with different probability distributions. To address this, Pang et al.^20^ introduced the concept of PLTS to tackle such issues.

#### Definition 1

Given a linguistic term set $S = \left\{ {s_{ - \tau } , \ldots ,s_{ - 1} ,s_{0} ,s_{1} , \ldots ,s_{\tau } } \right\}$, the PLTS can be defined as:

1 $$L(p) = \left\{ {L^{(k)} \left( {p^{(k)} } \right)|L^{(k)} \in S,p^{(k)} \ge 0,k = 1,2, \ldots ,\# L(p),\mathop \sum \limits_{k = 1}^{\# L(p)} p^{(k)} \le 1} \right\}$$

where $L^{(k)} \left( {p^{(k)} } \right)$ represents the linguistic term $L^{(k)}$ associated with probability $p^{(k)}$, $\# L(p)$ denotes the total number of linguistic terms in set $L(p)$. If $\sum\nolimits_{k = 1}^{{{{\# }}L(p)}} {p^{(k)} } = 1$, it signifies that complete probability distribution information for all possible linguistic terms is available. If $\sum\nolimits_{k = 1}^{{{{\# }}L(p)}} {p^{(k)} } < 1$, it indicates that the expert’s knowledge is insufficient to provide a complete assessment, and the probability distribution information for some linguistic terms is unknown.

To facilitate effective computations and address the issue of missing linguistic probability information, Pang et al.^20^ further introduced the concept of probabilistic information standardization.

#### Definition 2

Assuming a probabilistic linguistic term set $L(p)$ with its probability distribution $\sum\nolimits_{k = 1}^{\# L(p)} {p^{(k)} < 1}$, its standardized probabilistic linguistic term set $\dot{L}(p)$ is defined as:

2 $$\dot{L}(p) = \left\{ {L^{(k)} \left( {\dot{p}^{(k)} } \right)|k = 1,2, \ldots ,\# L(p)} \right\}$$

where $\dot{p}^{(k)} = p^{(k)} /\sum\nolimits_{k = 1}^{\# L(p)} {p^{(k)} }$, for all $k = 1,2, \ldots ,\# L(p)$.

After introducing the concept of the probabilistic linguistic term set, a scoring function for comparing different probabilistic linguistic term sets is also provided by Pang et al.^20^:

#### Definition 3

Let $L(p) = \left\{ {L^{(k)} \left( {p^{(k)} } \right)|k = 1,2, \ldots ,\# L(p)} \right\}$ be a probabilistic linguistic term set, $r^{(k)}$ be the subscript of linguistic term $L^{(k)}$, then the scoring function of $L(p)$ is defined as:

3 $$S(L(p)) = s_{{\sum\nolimits_{k = 1}^{\# L(p)} {r^{(k)} p^{(k)} } /\sum\nolimits_{k = 1}^{\# L(p)} {p^{(k)} } }} .$$

While it can be observed that the scoring function defined by Pang et al.^20^ allows for the comparison of different probabilistic linguistic term sets, its ultimate result remains a linguistic term, which may not be conducive to flexible calculations between probabilistic linguistic term sets. To address this issue, Wu et al.^22^ introduced the concept of the expectation function for probabilistic linguistic term set.

#### Definition 4

Given a linguistic term set $S = \left\{ {s_{\alpha } |\alpha = - \tau , \ldots , - 1,0,1, \ldots ,\tau } \right\}$, for a probabilistic linguistic term set $L(p) = \left\{ {L^{(k)} \left( {p^{(k)} } \right)|k = 1,2, \ldots ,\# L(p)} \right\}$, where $\alpha^{(k)}$ is the subscript of its linguistic term $s_{\alpha }^{(k)}$, the expectation value function of $L(p)$ is defined as:

4 $$E(L(p)) = \mathop \sum \limits_{k = 1}^{\# L(p)} \left( {\frac{{\alpha^{(k)} + \tau }}{2\tau }p^{(k)} } \right)/\mathop \sum \limits_{k = 1}^{\# L(p)} p^{(k)}$$

### Classical MACBETH method

Bana e Costa and Vansnick introduced the MACBETH (Measuring Attractiveness by a Categorical Based Evaluation Technique) method in 1990^23^. This method is a quantitative multi-criteria evaluation model aimed at establishing values. It effectively avoids requiring decision-makers to express their preferences directly using numerical values. In other words, this flexible method is not only applicable to criteria with quantitative measures but also suitable for assessing and ranking alternative solutions with qualitative criteria. Due to its simplicity and efficiency, this technology has been widely applied in various contexts in recent years, such as the evaluation of healthcare service systems and the design of risk matrices^24^.

The basic principles and specific steps of the MACBETH method are as follows:

*Step 1* Collect evaluation information $r_{ij}$ for each alternative $A_{i} (i = 1, \cdots ,m)$ under each criterion $C_{j} (j = 1, \cdots ,n)$ to ultimately obtain a decision matrix $D\left( {r_{ij} } \right)_{m \times n}$.

5 $$D\left( {r_{ij} } \right)_{m \times n} = \left[ {\begin{array}{*{20}c} {r_{11} } & \cdots & {r_{1j} } & \cdots & {r_{1n} } \\ \vdots & \ddots & \vdots & \ddots & \vdots \\ {r_{i1} } & \cdots & {r_{ij} } & \cdots & {r_{in} } \\ \vdots & \ddots & \vdots & \ddots & \vdots \\ {r_{m1} } & \cdots & {r_{mj} } & \cdots & {r_{mn} } \\ \end{array} } \right]_{m \times n} ,\quad i = 1, \ldots ,m,\quad j = 1, \ldots ,n$$

*Step 2* Obtain weight $w = (w_{1} ,w_{2} , \ldots ,w_{n} )$ for each criterion from the decision maker.

*Step 3* Convert semantic scales into numerical scales. Depending on the type of criteria (benefit and cost), each semantic scale is transformed into a consistent numerical scale, such as common five-level, seven-level, and nine-level semantic gradations. Taking a seven-level semantic scale as an example, let *S* be a linguistic evaluation set, it can be represented as* S* = {*s*_-3_: very poor, *s*_-2_: poor, *s*_-1_: somewhat poor,* s*_0_: fair, *s*_1_: somewhat good, *s*_2_: good, *s*_3_: very good}. The equivalent numerical grading for benefit criteria can be represented as *N* = {0, 1, 2, 3, 4, 5, 6}; and for cost criteria, the equivalent numerical grading is *N* = {6, 5, 4, 3, 2, 1, 0}.

*Step 4* Calculate the positive and negative reference standard values. Determine the positive and negative reference standard values for each criterion based on Eqs. (6) and (7).

6 $$r_{j}^{ - } = min \, r_{ij} ,\quad i = 1, \ldots ,m,\quad j = 1, \ldots ,n$$

7 $$r_{j}^{ + } = max \, r_{ij} ,\quad i = 1, \ldots ,m,\quad j = 1, \ldots ,n$$

*Step 5* Calculate the MACBETH score for each criterion.

8 $$v\left( {r_{ij} } \right) = v\left( {r_{j}^{ - } } \right) + \frac{{\left( {r_{ij} - r_{j}^{ - } } \right)}}{{\left( {r_{j}^{ + } - r_{j}^{ - } } \right)}}\left[ {v\left( {r_{j}^{ + } } \right) - v\left( {r_{j}^{ - } } \right)} \right],\quad i = 1, \ldots ,m, \, j = 1, \ldots ,n$$

where the values of $v\left( {r_{j}^{ - } } \right)$ and $v\left( {r_{j}^{ + } } \right)$ are typically 0 and 100, respectively.

*Step 6* Calculate the overall score *S*. Obtain the overall score $S_{i}$ for each alternative according to Eq. (9).

9 $$S_{i} = \mathop \sum \limits_{j = 1}^{n} v\left( {r_{ij} } \right) \cdot w_{j} ,\quad i = 1, \ldots ,m$$

*Step 7* Arrange the alternative solutions in descending order based on the total score $S_{i}$, thereby obtaining the final ranking of the alternative solutions.

## Methodology

**Fig. 1:**
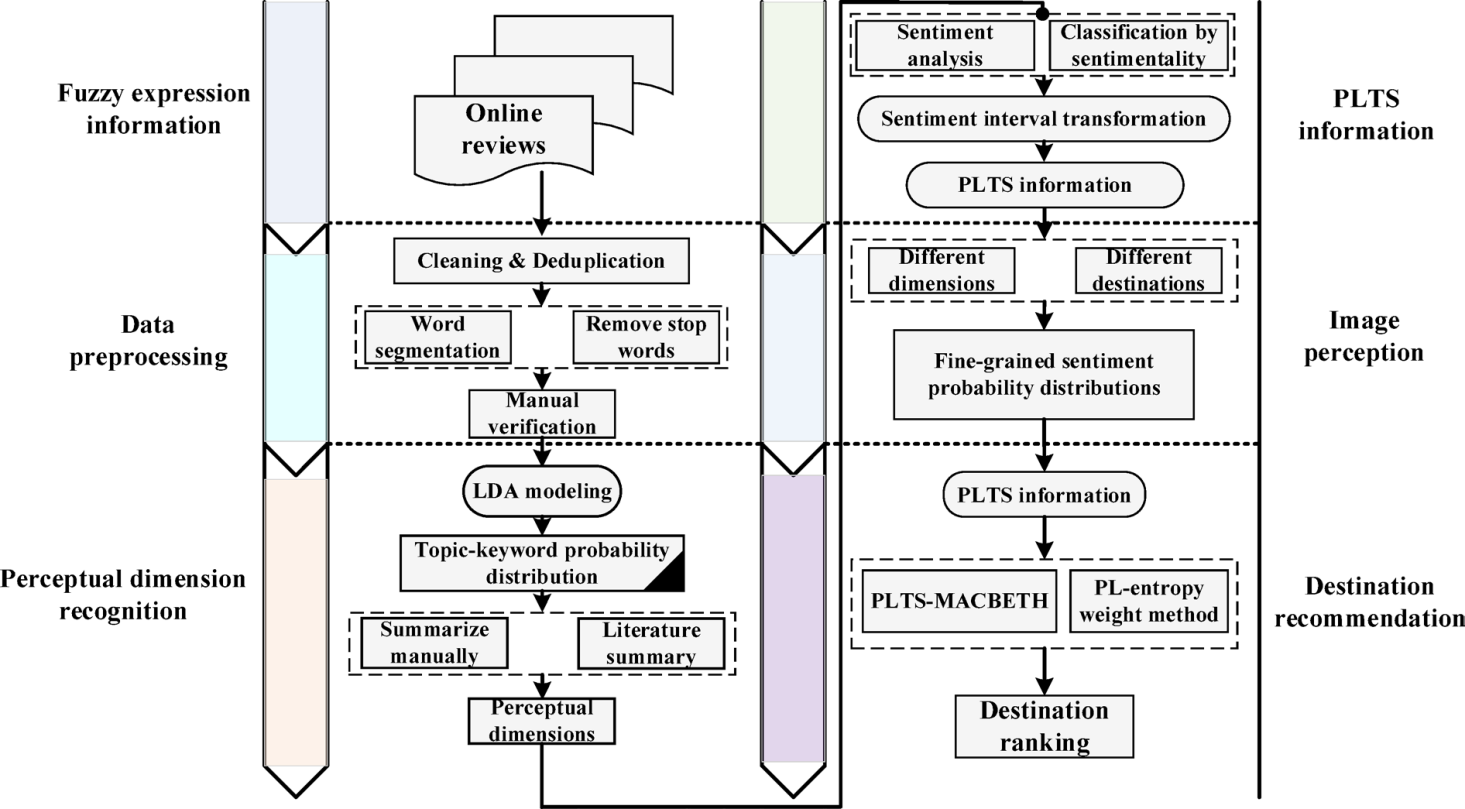
Research framework of this study.

Due to limited cognition and knowledge, combined with incomplete and uncertain information gathering, travelers often cannot comprehensively perceive the multidimensional image of a tourist destination. As a result, they may waver between several alternative tourist destinations. To address this issue, this study proposes a comprehensive decision support process. It is based on a vast amount of online reviews from travelers. It utilizes the LDA topic extraction model to identify the dimensions of traveler perception. Through sentiment analysis techniques and the theory of probabilistic linguistic term set, it finely characterizes the uncertainty and fuzziness in travelers’ online expressions, effectively constructing the destination image driven by fuzzy traveler expressions. This approach provides a multidimensional revelation of the real conditions of tourist destinations. Finally, the PL-MACBETH method is employed to harness collective intelligence for evaluating various tourist destinations, assisting potential travelers in making the best travel decisions. The specific solution is illustrated in Fig. 1.

### Data collection and preprocessing

To ensure the rapid and efficient acquisition of a substantial volume of authentic online reviews, this study utilized the Octopus collector tool to crawl and collect source data from well-known domestic online travel websites in China, such as Qunar, Tuniu, and Mafengwo, among others. The collected data was stored in CSV format for further analysis. Given the inherent variability in the length of original data obtained from web pages and the presence of empty lines and duplicated review data, post-crawling preprocessing was carried out. Initially, the Python programming language was employed to perform data cleaning operations, including removing empty lines and duplicates. Simultaneously, it was recognized that reviews with very few words often contain limited informative content, leading to the exclusion of review data with less than 10 words. Subsequently, the Jieba Chinese text segmentation tool and a stopword list were applied to tokenize the data and eliminate meaningless stop words. Lastly, a manual review process was employed to randomly inspect the data for potential anomalies, ensuring the overall validity of the final dataset. As this subsection describes the general data acquisition and preprocessing workflow, detailed case-specific information regarding data volume, collection period, destination types, and final sample composition is presented in “Case description and data processing” section.

### Identification of tourist perceptual dimensions based on LDA

To perform a multidimensional perception and evaluation of a destination image, it is essential to extract these dimensions from a large volume of online tourist reviews. To achieve this objective, this study employs the currently mature LDA topic extraction technique to identify tourist perceptual dimensions (e.g., Shashank and Behera^25^, Zhao and Huang^26^ and Zheng et al.^27^). In this study, “multidimensional” specifically refers to the representation of destination image across multiple latent perceptual dimensions extracted from tourists’ online reviews, including service, transportation, experience, history, and attractions. Each dimension is further characterized by probabilistic linguistic sentiment distributions, thereby enabling a more fine-grained and structured representation of destination conditions.

LDA is a probabilistic model and a technique used in natural language processing and machine learning. It was introduced by David Blei, Andrew Ng, and Michael Jordan in 2003^28^. LDA is primarily used for topic modeling, which is a method for discovering abstract topics within a collection of documents, such as a set of articles, reviews, or other text data. The key idea behind LDA is to represent documents as mixtures of topics and words as mixtures of topics in a corpus. It assumes that documents are generated by a probabilistic process where each document is a mixture of various topics, and each topic is a mixture of various words. LDA attempts to infer these topic distributions from the text data.

The outcome of running LDA is a set of topics, each associated with a distribution of words, and for each document, a distribution over topics. This makes LDA a valuable tool for automatically identifying and summarizing the main themes or topics within a collection of texts. It has various applications, including content recommendation, information retrieval, and text analysis in fields like marketing, academic research, and data mining. Based on this, the probability formula for the LDA model is as shown in Eq. (10).

10 $$p(\theta ,z,w|\alpha ,\beta ) = p(\theta |\alpha )\mathop \prod \nolimits p\left( {z_{n} |\theta } \right)p\left( {w_{n} |z_{n} ,\beta } \right)$$

where $w$ represents a word; $z$ is the topic of word $w$ in the document; $\theta$ follows a Dirichlet distribution with parameter $\alpha$, determining the topic distribution of the document; $\beta$ is the Dirichlet prior parameter, recording the probability of generating a specific word under a particular topic. In the generation process of the LDA model for a document, the topic distribution $\theta$ is determined by the Dirichlet ($\alpha$), and the number of words *N* is determined by a Poisson distribution. For each of the *N* words, the topic $z$ is chosen by a multinomial distribution with parameter value $\theta$, while the word $w$ is chosen based on multinomial distributions conditioned on $z$ and $\beta$.

After inputting the preprocessed textual data into the LDA topic model and defining the number of topics, denoted as *K*, the probability distribution of topics and their corresponding feature words is derived. Based on the results of the topic-feature word probability distribution, a careful categorization and analysis of the meaning and associated probabilities of feature words under each topic are performed. Concurrently, a concise summary is created by synthesizing existing literature on each topic. This process ultimately leads to the identification of the primary dimensions of tourist perception. At this stage, this study successfully extracts the dimensions of tourist perception and assigns each user online review to its respective topic.

### Information extraction of PLTS based on sentiment analysis

Tourist online reviews contain complex emotional nuances and sentiments. Therefore, the application of sentiment analysis techniques to extract valuable information from these reviews enables a quick and effective understanding of the multi-dimensional attitudes and acceptance of the public towards a tourist destination entity^29^. In recent years, numerous studies have focused on capturing the perceived destination image, controlling tourists’ emotional attitudes and overall impressions of the attractions they have experienced, utilizing web review data and sentiment analysis techniques. However, most existing studies predominantly categorize tourist destination emotional characteristics into coarse-grained positive, neutral, and negative sentiments based on sentiment polarity in review data. While such approaches are effective for capturing general emotional tendencies, their relatively limited granularity may constrain the fine-grained representation of tourists’ heterogeneous evaluations across different destination dimensions. In addition, although polarity-based sentiment classification provides a useful foundation for emotional analysis, it does not explicitly model the probability distribution and uncertainty structure embedded in tourists’ linguistic expressions. To address this limitation, this study further introduces PLTS to transform sentiment values into probabilistic multi-granularity linguistic assessments, thereby enabling a more refined representation of evaluative ambiguity and uncertainty^30^. To address this limitation, this study introduces PLTS to effectively portray sentiment tendencies and their probability distribution information in tourist online reviews^31^. This transformation allows for the conversion of qualitative evaluations by tourists into quantitative assessment data, thereby facilitating the extraction of PLTS information.

**Fig. 2:**
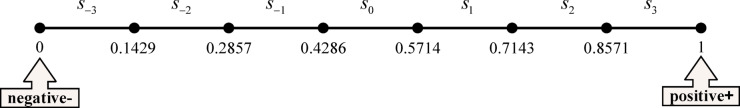
Mutual transformation relationship between PLTS $S = \left\{ {s_{\alpha } |\alpha = - 3, - 2, - 1,0,1,2,3} \right\}$ and the sentiment value intervals.

First, this study employs the Chinese sentiment analysis tool SnowNLP to calculate the sentiment tendencies for each review data under different topics in the previous process. It is noteworthy that SnowNLP is a Python library developed, inspired by the English sentiment analysis tool TextBlob, which greatly facilitates the exploration and processing of Chinese text content. Specifically, SnowNLP can perform functions such as Chinese word segmentation, sentiment analysis, part-of-speech tagging, and text classification. In general, sentiment values computed by SnowNLP fluctuate within the range of [0, 1], where 0 represents entirely negative sentiment, and 1 signifies completely positive sentiment. This numerical scale aligns precisely with the PLTS. Subsequently, this study classifies the degree of sentiment and divides the sentiment values into different levels according to the needs of different granularity, such as 5 levels, 7 levels, or even 9 levels. Taking 7 levels as an example, Fig. 2 clearly illustrates the mutual transformation relationship between PLTS $S = \left\{ {s_{\alpha } |\alpha = - 3, - 2, - 1,0,1,2,3} \right\}$ and the sentiment value intervals.

Next, the probability calculation formula for each transformed linguistic term is as follows:

11 $$P_{i\alpha }^{j} = \frac{{N_{i\alpha }^{j} }}{{\mathop \sum \nolimits_{k = 1}^{{{{\# }}L(p)}} N_{i\alpha }^{j} }}$$

where $P_{i\alpha }^{j}$ represents the probability of linguistic term $s_{\alpha }$ in topic $C_{j}$ for the alternative destination $A_{i}$, $N_{i\alpha }^{j}$ represents the number of tourist online reviews contained in linguistic term $s_{\alpha }$ for the alternative destination $A_{i}$ in topic $C_{j}$, and ${{\# }}L(p)$ represents the total number of linguistic terms.

Following the above processing procedure, the PLTS information within the tourist online review data is effectively extracted and represented.

### Perceived destination image driven by PLTS information

Given that PLTS can not only represent linguistic evaluative information but also encapsulate the importance of linguistic evaluations in the form of probability distributions, the construction of the perceived destination image under the PLTS information has several advantages^32^. On the one hand, it allows for a granular representation of the actual operational status of tourist destinations. On the other hand, it employs linguistic scales that are in accordance with real-world significance, depicting the image of tourist destinations in a manner more aligned with people’s cognitive norms and thought processes. In the previous phase, PLTS information extracted from online tourist reviews, including linguistic evaluative information and their corresponding probability distribution information, has already been collected. In this subsection, a data analysis and visualization approach is applied to different dimensions (i.e., topics) within various tourist destinations. This results in fine-grained probability distribution of sentiment tendencies for each dimension of tourist destinations. Consequently, it provides an intuitive means to comprehensively grasp the perceived destination image by tourists and the multidimensional distribution and differences among these images.

### Tourist destination recommendation based on PL-MACBETH approach

Recommendations for tourist destinations can be represented as an investigation and ranking of available tourist destinations using key dimensional features, thereby modeling it as a criteria decision problem. Simultaneously, as tourist destinations become more diverse and tourism elements more complex, the decision-making process for potential travelers has evolved from a single-criterion decision-making problem to a MCDM problem^27,33^. To address this, this study extends the classical MACBETH method, which is both straightforward and practical, to the probabilistic linguistic context. In conjunction with the expectation value function of PLTS, it introduces the PL-MACBETH decision method. Finally, using the probabilistic linguistic decision information outlined above, the PL-entropy weight method is introduced to calculate the objective weights for each criterion. The PL-MACBETH method is then applied to rank different tourist destinations, with the aim of providing decision support for prospective travelers. It is worth noting that the original probabilistic linguistic decision information obtained through sentiment analysis already takes into account the criterion’s category, and therefore, this aspect will not be emphasized further in the subsequent steps. The specific steps are as follows:

*Step 1* Calculate the probabilistic linguistic assessment information $L_{ij} (p)$ for each criterion $C_{j} (j = 1, \cdots ,n)$ under each alternative destination $A_{i} (i = 1, \cdots ,m)$, resulting in the eventual construction of probabilistic linguistic decision matrix $R = \left[ {L_{ij} (p)} \right]_{m \times n}$.

12 $$R = \left[ {L_{ij} (p)} \right]_{m \times n} = \left[ {\begin{array}{*{20}c} {L_{11} (p)} & {L_{12} (p)} & \cdots & {L_{1n} (p)} \\ {L_{21} (p)} & {L_{22} (p)} & \cdots & {L_{2n} (p)} \\ \vdots & \vdots & \ddots & \vdots \\ {L_{m1} (p)} & {L_{m2} (p)} & \cdots & {L_{mn} (p)} \\ \end{array} } \right]_{m \times n} ,\quad i = 1, \ldots ,m,\quad j = 1, \ldots ,n$$

*Step 2* Employ the PL-entropy weight method to compute the weights $w = (w_{1} ,w_{2} , \ldots ,w_{n} )$ of each criterion. Initially, utilize Eq. (13) to calculate the information entropy for each PLTS.

13 $$E(L_{ij} (p)) = - \frac{1}{{\log_{2} {{\# }}L_{ij} (p)}}\mathop \sum \limits_{k = 1}^{{{{\# }}L_{ij} (p)}} p_{ij}^{(k)} \log_{2} p_{ij}^{(k)}$$

Next, calculate the average entropy $\overline{E} (C_{j} )$ under each criterion $C_{j}$, as follows:

14 $$\overline{E} (C_{j} ) = \frac{1}{m}\mathop \sum \limits_{i = 1}^{m} E(L_{j} (p))$$

Finally, obtain the weights $w_{j}$ for each criterion, as follows:

15 $$w_{j} = \frac{{1 - \overline{E} (C_{j} )}}{{\mathop \sum \nolimits_{i = 1}^{m} \left( {1 - \overline{E} (C_{j} )} \right)}},\;j = 1, \ldots ,n$$

*Step 3* Calculate the expectation values for each PLTS using Eq. (4).

*Step 4* Rank the alternative tourist destinations in accordance with the sorting procedure outlined in the classic MACBETH method, which involves Eqs. (6) to (9).

## Case study

### Case description and data processing

To empirically validate the effectiveness of the proposed framework in destination image construction and recommendation, this study selected Zhejiang Province, China, as the case context due to its rich tourism resources and the diversity of high-quality tourist destinations. Among the officially recognized 5A-level tourist attractions in Zhejiang Province, four representative destinations were selected as alternative cases after preliminary screening: West Lake Scenic Area in Hangzhou (A1), South Lake Tourist Area in Jiaxing (A2), Tianyi Pavilion Moon Lake Scenic Area in Ningbo (A3), and Thousand Island Lake Scenic Area in Hangzhou (A4). These destinations represent heterogeneous tourism contexts, including natural scenic attractions, historical-cultural heritage destinations, and integrated leisure tourism areas, thus providing an appropriate empirical setting for testing the robustness and applicability of the proposed probabilistic linguistic decision framework.

For collecting tourist online review data, to ensure the fairness and authenticity of the evaluation information, this study selected the official website of Qunar, a well-known online travel platform in China. Qunar is a leading travel search engine in China and the world’s largest Chinese online travel website. It aims to provide real-time searches for flights, hotels, venues, vacation products, and offers group buying services for travel products as well as other travel information services. Next, the Octopus web scraping tool was used to real-time crawl online review data for the four tourist destinations. To maintain the consistency of the review data volume for each destination, 1500 reviews were crawled for each attraction in preparation for subsequent work, resulting in an initial dataset of 6000 raw online reviews. The review data were collected during the period from January 2024 to December 2025. Following the preprocessing steps outlined in “Data collection and preprocessing” section, the source data underwent cleaning, deduplication, and removal of reviews with a word count less than 10. It is worth noting that during the inspection of the original data, numerous high-frequency vocabulary related to place names such as “Hangzhou”, “Jiaxing” and “Ningbo” as well as some lengthy but non-substantive reviews like “good, good, good, good, good…” and “nice, nice, nice, nice…” were identified. Such reviews, in part, would introduce noise to the subsequent topic extraction and destination recommendation processes. Therefore, these reviews were uniformly removed in this study. As a result of this processing, an uneven volume of online review data for the four tourist destinations was observed. To address this, the study selected 1000 valid reviews from the preprocessed data for each destination, yielding a balanced final sample of 4000 valid reviews for subsequent analysis. These selected reviews were then subjected to segmentation and removal of meaningless stopwords using the jieba Chinese word segmentation tool, followed by stop-word filtering and normalization of textual expressions, resulting in the final dataset consisting of 4000 reviews. Lastly, a secondary manual inspection was conducted to ensure the accuracy of the original data.

It should be noted that the manual inspection step in this study is introduced solely as a lightweight data quality assurance procedure to ensure the semantic validity of a small subset of noisy or non-informative reviews. This step does not form part of the core algorithmic framework and does not affect the general applicability or automation potential of the proposed method. In practical deployment, this procedure can be fully replaced by automated filtering techniques such as rule-based filtering or pretrained language models for noise detection.

### Multidimensional perceived destination image driven by tourists’ fuzzy expression information

When employing the LDA algorithm to extract topics from the case data, it is necessary to manually set the number of topics, denoted as *K*. Considering that excessively fine-grained divisions of tourist perception dimensions would increase the complexity of the subsequent recommendation process, and an overly dense number of dimensions can compromise their independence and reduce their low relevance. Therefore, this study, drawing from existing research literature^34^, set the number of topics *K* to 5, 6, 7, 8, 9, and 10, respectively. In the LDA implementation, the Dirichlet hyperparameters α and β were set using a symmetric configuration to ensure balanced topic and word distributions, which is commonly adopted in short-text topic modeling studies.

Then, we incorporated these values into the LDA topic model to observe the meaning and probability distribution of various characteristic words under different topics. To ensure model quality, we further computed topic coherence scores (CV coherence) for each candidate K value to quantitatively evaluate semantic consistency among topics. Furthermore, we employed auxiliary quantitative coherence metrics to assess the coherence of the topic model. Through iterative comparisons and experiments, it was found that when the number of topics *K* was set to 5, not only did the meanings of characteristic words under each topic exhibit high distinctiveness and identifiability, but the probability distribution of characteristic words was also relatively high. Specifically, K = 5 achieved the highest coherence score among all tested configurations, and also provided the most interpretable topic structure in terms of semantic separation. This facilitated the categorization and summarization of different dimensions of tourist perception. Therefore, this study ultimately set the number of topics* K* to 5 and identified five major tourist perception dimensions within the case data, namely, Topic 1: Service, Topic 2: Transportation, Topic 3: Experience, Topic 4: History, and Topic 5: Attractions. Topic labeling was conducted based on the top 10 keywords (Top-N = 10) with the highest probability within each topic, combined with manual semantic interpretation to ensure interpretability and consistency. Table 1 provides a detailed list of the top 10 weighted words with probability values under each topic, representing the concretization of different dimensions of tourist perception. Each review was assigned to the topic with the highest posterior probability generated by the LDA model, ensuring a clear mapping between textual data and latent topics. Thus, the multidimensional structure of destination image in this study is operationalized through five latent topics identified via LDA.

**Table 1:** The top 10 weighted words and probability values under each topic.

Service		Transportation		Experience		History		Attractions	
Good	0.066	Cruises	0.015	Space	0.015	Worthwhile	0.032	Nature	0.035
Convenient	0.058	Bridge	0.015	Period	0.014	History	0.028	Fine	0.033
Very	0.049	Sights	0.015	Courier	0.014	Park	0.024	Tourism	0.030
Scenery	0.038	Jetty	0.015	None	0.013	Gangnam	0.016	Sensory	0.017
Ticketing	0.027	Ticket	0.014	Well	0.013	Culture	0.016	Territory	0.014
Worthwhile	0.018	Handy	0.009	Hours	0.013	Sights	0.015	Specially	0.013
Service	0.014	Time	0.009	View	0.012	Library	0.015	Favor	0.012
Fast	0.012	Direct	0.009	Island	0.011	Ancient	0.014	Much	0.011
Lump	0.012	Hour	0.009	Feeling	0.011	Museums	0.013	Interior	0.011
Somewhat	0.012	Ferry	0.008	Fairly	0.009	Place	0.013	Grand	0.010

From Table 1, it is evident that each topic corresponds to distinct aspects of tourists’ perceptions regarding the tourist destination. Under the “Service” topic, the high-probability characteristic words such as “good”, “convenient” and “service” signify tourists’ favorable perceptions of the services offered by the destination. In the “Transportation” topic, words like “cruises”, “handy” and “time” indicate tourists’ opinions on the transportation facilities within and around the destination, highlighting aspects such as convenience and accessibility. Moving on to the “Experience” topic, terms like “courier”, “well” and “feeling” reflect tourists’ assessments of the overall tourism experience, encompassing factors like the quality of hospitality and the ambiance provided by the destination. In the “History” topic, words such as “history”, “culture” and “worthwhile” underscore tourists’ recognition of the historical significance and cultural richness of the destination, suggesting an appreciation for its heritage. Lastly, the “Attractions” topic is characterized by terms like “nature”, “favor” and “grand”, which point towards tourists’ perceptions of the natural beauty and grandeur of the destination’s main attractions. By examining the high-probability characteristic words associated with each topic, we gain insights into the diverse facets of tourists’ perceptions, ranging from service quality and transportation convenience to cultural heritage and natural attractions, thereby providing a comprehensive understanding of the destination’s appeal.

After identifying the tourist perception dimension system, each piece of tourist review data was automatically categorized into their respective topics. There were 840 relevant review data in Topic 1 (Service), 584 in Topic 2 (Transportation), 877 in Topic 3 (Experience), 874 in Topic 4 (History), and 825 in Topic 5 (Attractions). Subsequently, the Chinese sentiment analysis tool, SnowNLP, was used to calculate the sentiment values for each review in each topic. A seven-scale linguistic term set, *S* = {*s*_-3_: very poor, *s*_-2_: poor, *s*_-1_: below average, *s*_0_: average, *s*_1_: above average, *s*_2_: good, *s*_3_: very good}, was employed. Following the transformation rules and calculation formula (11) outlined in “Information extraction of PLTS based on sentiment analysis” section, the multitude of emotional information from tourist perceptions was transformed into PLTS information, including linguistic evaluations and their corresponding probability distributions (calculated results were rounded to four decimal places). The sentiment polarity scores generated by SnowNLP, ranging from 0 to 1, were transformed into seven linguistic terms within the PLTS framework using a structured thresholding strategy. Specifically, an equal-interval segmentation approach was adopted to divide the sentiment range into seven linguistic levels, ensuring consistency and interpretability of the mapping process. The seven linguistic terms correspond to progressively increasing sentiment intensity from extremely negative to extremely positive. This transformation strategy is widely adopted in linguistic decision-making studies and ensures compatibility between numerical sentiment outputs and linguistic representation structures.

**Fig. 3:**
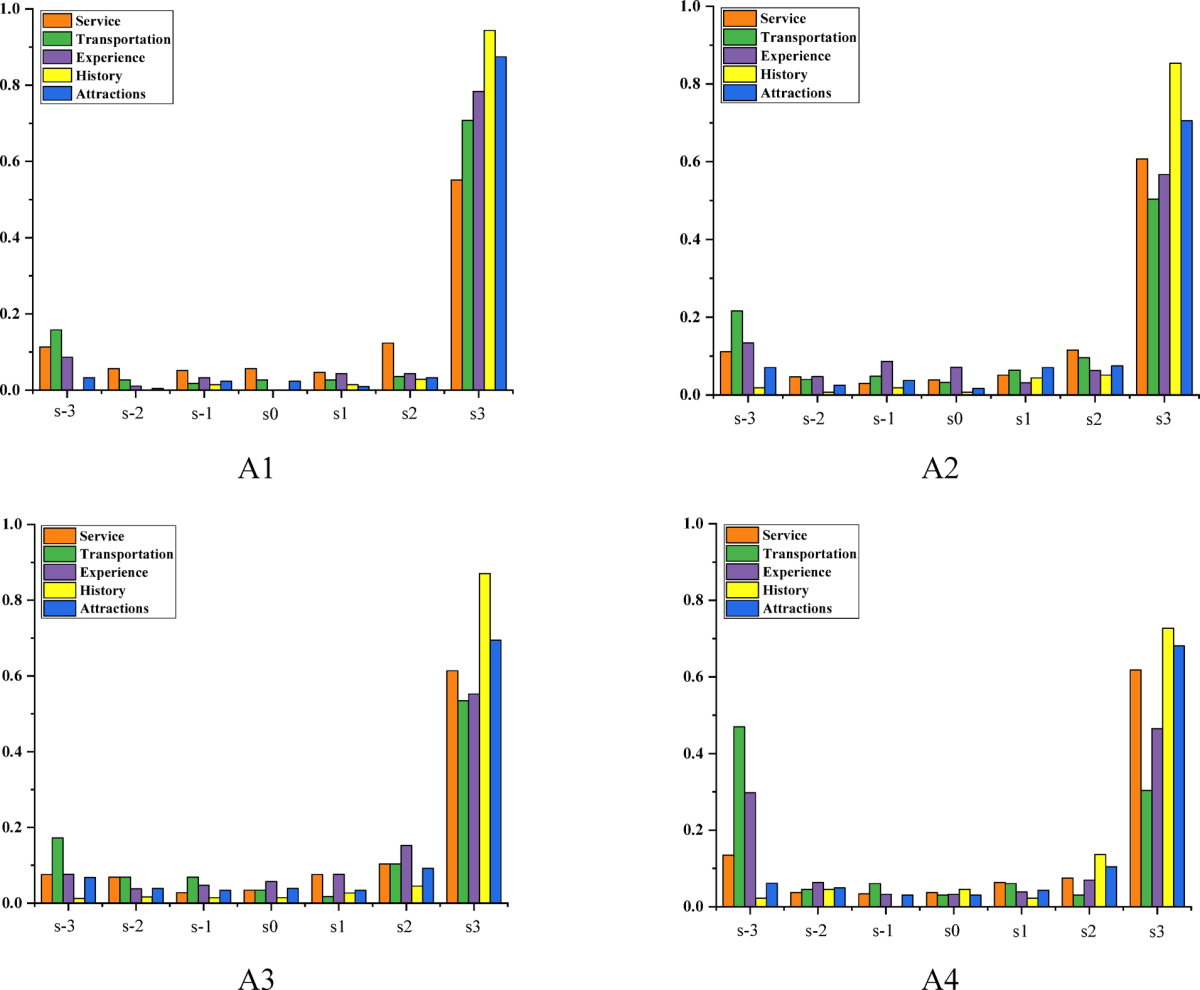
Probability distribution of the seven-grain emotional tendency of the perceived destination image. West Lake Scenic Area in Hangzhou (A1), South Lake Tourist Area in Jiaxing (A2), Tianyi Pavilion Moon Lake Scenic Area in Ningbo (A3), and Thousand Island Lake Scenic Area in Hangzhou (A4).

Utilizing these PLTS information, Fig. 3 visually presents the probability distributions of emotional tendencies at seven granularities within each dimension for the four tourist destinations, thereby allowing for a rapid perception of the multidimensional image of these destinations. From Fig. 3, it is evident that in most cases, tourists’ probabilities of giving “very good” evaluations under the five dimensions are significantly higher than other evaluation terms, with the exception of the Thousand Island Lake Scenic Area. Taking the “Transportation” dimension of the Thousand Island Lake Scenic Area as an example, the probability of tourists giving a “very poor” evaluation is as high as 0.4697, indicating that there is a pressing need for improvement in transportation aspects for this destination. Simultaneously, the performance in the “Experience” dimension is also unsatisfactory, with a probability of 0.2978 for tourists giving an “very poor” evaluation. However, in the “Service” dimension, the Thousand Island Lake Scenic Area performed the best with a high probability of 0.6180 for tourists giving an “very good” evaluation. Under the “very good” evaluation term, whether in the “Transportation” and “Experience” dimensions or the “History” and “Attractions” dimensions, the West Lake Scenic Area ranks first. The probability distributions of other evaluation terms, such as “poor”, “below average”, “average”, “above average” and “good” are intuitively represented in the graph.

### Destination recommendation

In order to determine the optimal tourist destination, we utilize the PL-MACBETH decision-making method and the PL-entropy weight method based on the PLTS information described above. Following the algorithmic steps recommended in “Tourist destination recommendation based on PL-MACBETH approach” section, we evaluate four different tourist destinations across five dimensions: Service (C1), Transportation (C2), Experience (C3), History (C4), and Attractions (C5). Firstly, the detailed distribution of the number of reviews corresponding to sentiment analysis-transformed linguistic evaluation terms is presented in Table 2. Subsequently, according to Eq. (11), it is further transformed into a probabilistic linguistic decision matrix, as shown in Table 3. Since the probability distributions for each PLTS sum up to 1, there is no need for standardization.

**Table 2:** The results of sentiment analysis-transformed linguistic evaluation terms.

	*A*_1_	*A*_2_
*C*_1_	− 3_(22)_, − 2_(11)_, − 1_(10)_, 0_(11)_, 1_(9)_, 2_(24)_, 3_(107)_	− 3_(26)_, − 2_(11)_, − 1_(7)_, 0_(9)_, 1_(12)_, 2_(27)_, 3_(142)_
*C*_2_	− 3_(53)_, − 2_(9)_, − 1_(6)_, 0_(9)_, 1_(9)_, 2_(12)_, 3_(237)_	− 3_(27)_, − 2_(5)_, − 1_(6)_, 0_(4)_, 1_(8)_, 2_(12)_, 3_(63)_
*C*_3_	− 3_(16)_, − 2_(2)_, − 1_(6)_, 0_(0)_, 1_(8)_, 2_(8)_, 3_(145)_	− 3_(17)_, − 2_(6)_, − 1_(11)_, 0_(9)_, 1_(4)_, 2_(8)_, 3_(72)_
*C*_4_	− 3_(0)_, − 2_(0)_, − 1_(1)_, 0_(0)_, 1_(1)_, 2_(2)_, 3_(67)_	− 3_(5)_, − 2_(2)_, − 1_(5)_, 0_(2)_, 1_(12)_, 2_(14)_, 3_(233)_
*C*_5_	− 3_(7)_, − 2_(1)_, − 1_(5)_, 0_(5)_, 1_(2)_, 2_(7)_, 3_(188)_	− 3_(17)_, − 2_(6)_, − 1_(9)_, 0_(4)_, 1_(17)_, 2_(18)_, 3_(170)_
	*A*_3_	*A*_4_
*C*_1_	− 3_(11)_, − 2_(10)_, − 1_(4)_, 0_(5)_, 1_(11)_, 2_(15)_, 3_(89)_	− 3_(36)_, − 2_(10)_, − 1_(9)_, 0_(10)_, 1_(17)_, 2_(20)_, 3_(165)_
*C*_2_	− 3_(10)_, − 2_(4)_, − 1_(4)_, 0_(2)_, 1_(1)_, 2_(6)_, 3_(31)_	− 3_(31)_, − 2_(3)_, − 1_(4)_, 0_(2)_, 1_(4)_, 2_(2)_, 3_(20)_
*C*_3_	− 3_(8)_, − 2_(4)_, − 1_(5)_, 0_(6)_, 1_(8)_, 2_(16)_, 3_(58)_	− 3_(137)_, − 2_(29)_, − 1_(15)_, 0_(15)_, 1_(18)_, 2_(32)_, 3_(214)_
*C*_4_	− 3_(6)_, − 2_(8)_, − 1_(7)_, 0_(7)_, 1_(13)_, 2_(22)_, 3_(423)_	− 3_(1)_, − 2_(2)_, − 1_(0)_, 0_(2)_, 1_(1)_, 2_(6)_, 3_(32)_
*C*_5_	− 3_(14)_, − 2_(8)_, − 1_(7)_, 0_(8)_, 1_(7)_, 2_(19)_, 3_(143)_	− 3_(10)_, − 2_(8)_, − 1_(5)_, 0_(5)_, 1_(7)_, 2_(17)_, 3_(111)_

**Table 3:** Probabilistic linguistic decision matrix for each alternative tourist destination.

	*A*_1_	*A*_2_
*C*_1_	{*s*_−3_(0.1134), *s*_−2_(0.0567), *s*_−1_(0.0515), *s*_0_(0.0567), *s*_1_(0.0464), *s*_2_(0.1237), *s*_3_(0.5515)}	{*s*_−3_(0.1111), *s*_−2_(0.0470), *s*_−1_(0.0299), *s*_0_(0.0385), *s*_1_(0.0513), *s*_2_(0.1154), *s*_3_(0.6068)}
*C*_2_	{*s*_−3_(0.1582), *s*_−2_(0.0269), *s*_−1_(0.0179), *s*_0_(0.0269), *s*_1_(0.0269), *s*_2_(0.0358), *s*_3_(0.7075)}	{*s*_−3_(0.2160), *s*_−2_(0.0400), *s*_−1_(0.0480), *s*_0_(0.0320), *s*_1_(0.0640), *s*_2_(0.0960), *s*_3_(0.5040)}
*C*_3_	{*s*_−3_(0.0865), *s*_−2_(0.0108), *s*_−1_(0.0324), *s*_1_(0.0432), *s*_2_(0.0432), *s*_3_(0.7838)}	{*s*_−3_(0.1339), *s*_−2_(0.0472), *s*_−1_(0.0866), *s*_0_(0.0709), *s*_1_(0.0315), *s*_2_(0.0630), *s*_3_(0.5669)}
*C*_4_	{*s*_−1_(0.0141), *s*_1_(0.0141), *s*_2_(0.0282), *s*_3_(0.9437)}	{*s*_−3_(0.0183), *s*_−2_(0.0073), *s*_−1_(0.0183), *s*_0_(0.0073), *s*_1_(0.0440), *s*_2_(0.0513), *s*_3_(0.8535)}
*C*_5_	{*s*_−3_(0.0326), *s*_−2_(0.0047), *s*_−1_(0.0233), *s*_0_(0.0233), *s*_1_(0.0093), *s*_2_(0.0326), *s*_3_(0.8744)}	{*s*_−3_(0.0705), *s*_−2_(0.0249), *s*_−1_(0.0373), *s*_0_(0.0166), *s*_1_(0.0705), *s*_2_(0.0747), *s*_3_(0.7054)}
	*A*_3_	*A*_4_
*C*_1_	{*s*_−3_(0.0759), *s*_−2_(0.0690), *s*_−1_(0.0276), *s*_0_(0.0345), *s*_1_(0.0759), *s*_2_(0.1034), *s*_3_(0.6138)}	{*s*_−3_(0.1348), *s*_−2_(0.0375), *s*_−1_(0.0337), *s*_0_(0.0375), *s*_1_(0.0637), *s*_2_(0.0749), *s*_3_(0.6180)}
*C*_2_	{*s*_−3_(0.1724), *s*_−2_(0.0690), *s*_−1_(0.0690), *s*_0_(0.0345), *s*_1_(0.0172), *s*_2_(0.1034), *s*_3_(0.5345)}	{*s*_−3_(0.4697), *s*_−2_(0.0455), *s*_−1_(0.0606), *s*_0_(0.0303), *s*_1_(0.0606), *s*_2_(0.0303), *s*_3_(0.3030)}
*C*_3_	{*s*_−3_(0.0762), *s*_−2_(0.0381), *s*_−1_(0.0476), *s*_0_(0.0571), *s*_1_(0.0762), *s*_2_(0.1524), *s*_3_(0.5524)}	{*s*_−3_(0.2978), *s*_−2_(0.0630), *s*_−1_(0.0326), *s*_0_(0.0326), *s*_1_(0.0391), *s*_2_(0.0696), *s*_3_(0.4652)}
*C*_4_	{*s*_−3_(0.0123), *s*_−2_(0.0165), *s*_−1_(0.0144), *s*_0_(0.0144), *s*_1_(0.0267), *s*_2_(0.0453), *s*_3_(0.8704)}	{*s*_−3_(0.0227), *s*_−2_(0.0455), *s*_0_(0.0455), *s*_1_(0.0227), *s*_2_(0.1364), *s*_3_(0.7273)}
*C*_5_	{*s*_−3_(0.0680), *s*_−2_(0.0388), *s*_−1_(0.0340), *s*_0_(0.0388), *s*_1_(0.0340), *s*_2_(0.0922), *s*_3_(0.6942)}	{*s*_−3_(0.0613), *s*_−2_(0.0491), *s*_−1_(0.0307), *s*_0_(0.0307), *s*_1_(0.0429), *s*_2_(0.1043), *s*_3_(0.6810)}

Subsequently, we apply Eq. (13) through Eq. (15) to calculate the information entropy $E(L_{ij} (p)) \, (i = 1, \ldots ,4;j = 1, \ldots ,5)$ for each PLTS and the respective criteria weights $w_{j} (j = 1, \ldots ,5)$, as depicted in Tables 4 and 5.

**Table 4:** Information entropy for each PLTS.

	*C*_1_	*C*_2_	*C*_3_	*C*_4_	*C*_5_
*A*_1_	0.7474	0.5238	0.4288	0.1415	0.3000
*A*_2_	0.6798	0.7513	0.7285	0.3307	0.5636
*A*_3_	0.6810	0.7336	0.7399	0.3093	0.5849
*A*_4_	0.6667	0.7241	0.7331	0.4914	0.5990

**Table 5:** Weight of criteria.

Criteria	*C*_1_	*C*_2_	*C*_3_	*C*_4_	*C*_5_
$w_{i}$	0.1434	0.1484	0.1603	0.3193	0.2286

Based on Eq. (4), we calculate the expectation values for each PLTS, as presented in Table 6.

**Table 6:** Expectation value for each PLTS.

	*C*_1_	*C*_2_	*C*_3_	*C*_4_	*C*_5_
*A*_1_	0.7405	0.7791	0.8613	0.9812	0.9279
*A*_2_	0.7742	0.6653	0.7126	0.9365	0.8396
*A*_3_	0.7885	0.6839	0.7810	0.9407	0.8309
*A*_4_	0.7591	0.4116	0.5870	0.8864	0.8303

Following the ranking steps of the classical MACBETH method (i.e., Eq. (6) to Eq. (9)), we perform a further analysis and calculation on the data in Table 6. This leads to the determination of the positive and negative reference values for each criterion, MACBETH scores, and the overall scores for each alternative tourist destination (Tables 7, 8, 9).

**Table 7:** Values of the reference levels.

	*C*_1_	*C*_2_	*C*_3_	*C*_4_	*C*_5_
$r^{ - }$	0.7405	0.4116	0.5870	0.8864	0.8303
$r^{ + }$	0.7885	0.7791	0.8613	0.9812	0.9279

**Table 8:** The MACBETH scores.

	*C*_1_	*C*_2_	*C*_3_	*C*_4_	*C*_5_
*A*_1_	0	100	100	100	100
*A*_2_	70.2034	69.0409	45.8038	52.8630	9.5160
*A*_3_	100	74.0954	70.7228	57.2530	0.6558
*A*_4_	38.5799	0	0	0	0

**Table 9:** Overall evaluation scores for each alternative tourist destination.

	*C*_1_	*C*_2_	*C*_3_	*C*_4_	*C*_5_	*S*
*A*_1_	0	14.8357	16.0349	31.9273	22.8589	85.6568
*A*_2_	10.0694	10.2427	7.3446	16.8778	2.1753	46.7097
*A*_3_	14.3432	10.9926	11.3403	18.2794	0.1499	55.1053
*A*_4_	5.5336	0	0	0	0	5.5336

Finally, the overall scores $S_{i} \, (i = 1, \cdots ,4)$ for each alternative destination are sorted. Based on the results $S_{1} > S_{3} > S_{2} > S_{4}$, the order of alternative destinations $A_{1} \succ A_{3} \succ A_{2} \succ A_{4}$ is determined, with destination *A*_1_ being identified as the optimal tourist destination.

### Comparative analysis

#### Comparison with the existing methods

To assess the feasibility and effectiveness of the PL-MACBETH decision-making method, this subsection performs a comparative analysis with three other existing methods: the real-number environment MACBETH decision method, the intuitionistic fuzzy MACBETH (IF-MACBETH) decision method and the hesitant fuzzy linguistic term sets (HFLTS)-based MACBETH method (HFL-MACBETH). It is worth noting that to ensure comparability among the four methods, the criterion weights used in the case study described earlier are retained for the implementation of the other three methods. For the real-number environment MACBETH decision process, initially, the probabilistic linguistic decision information in Table 3 is converted into real-number decision information. Because the evaluation information for the linguistic terms “better”, “good” and “very good” all represent positive feedback from tourists, the probability information for these three terms is summed for each PLTS in the matrix. This results in the proportion of positive emotions for each dimension of each destination, constituting the initial decision matrix in the real-number environment, as shown in Table 10. Subsequently, the four tourist destinations are ranked following the specific steps of the classical MACBETH method.

**Table 10:** Initial decision matrix under real number environment for each alternative tourist destination.

	*C*_1_	*C*_2_	*C*_3_	*C*_4_	*C*_5_
*A*_1_	0.7405	0.7791	0.8613	0.9812	0.9279
*A*_2_	0.7742	0.6653	0.7126	0.9365	0.8396
*A*_3_	0.7885	0.6839	0.7810	0.9407	0.8309
*A*_4_	0.7591	0.4116	0.5870	0.8864	0.8303

For the IF-MACBETH decision process within a fuzzy environment, similarly, the initial step involves converting the probabilistic linguistic decision information from Table 3 into intuitionistic fuzzy decision information. Using the concepts of membership, hesitation, and non-membership in intuitionistic fuzzy sets, they correspond precisely to the probability information of the evaluation linguistic terms “above average”, “good” and “very good”; “average”; “below average”, “poor” and “very poor” in each PLTS, respectively. Consequently, the initial intuitionistic fuzzy decision matrix is obtained, as shown in Table 11.

**Table 11:** Intuitionistic fuzzy decision matrix for each alternative tourist destination.

	*C*_1_	*C*_2_	*C*_3_	*C*_4_	*C*_5_
*A*_1_	(0.7216, 0.2216)	(0.7701, 0.2030)	(0.8703, 0.1297)	(0.9859, 0.0141)	(0.9163, 0.0605)
*A*_2_	(0.7735, 0.1880)	(0.6640, 0.3040)	(0.6614, 0.2677)	(0.9487, 0.0440)	(0.8506, 0.1328)
*A*_3_	(0.7931, 0.1724)	(0.6552, 0.3103)	(0.7801, 0.1619)	(0.9424, 0.0432)	(0.8204, 0.1408)
*A*_4_	(0.7566, 0.2060)	(0.3939, 0.5758)	(0.5739, 0.3935)	(0.8864, 0.0682)	(0.8282, 0.1411)

Subsequently, in accordance with the score function of intuitionistic fuzzy sets $S(\alpha ) = \mu - \nu$, the scores for each intuitionistic fuzzy number $\alpha_{ij} = \left( {\mu_{ij} ,\nu_{ij} } \right)(i = 1, \ldots ,4;j = 1, \ldots ,5)$ in Table 11 are computed . Next, based on the decision matrix transformed by the score function, the four tourist destinations are ranked using the calculation steps outlined in Eqs. (6) to (9).

HFLTS has been widely recognized for its capability to model hesitation and linguistic uncertainty in expert evaluations. In the HFL-MACBETH implementation, the probabilistic linguistic information is first transformed into hesitant fuzzy linguistic representations following the standard aggregation of possible linguistic terms without probability distribution information. Subsequently, the classical MACBETH procedure is applied for ranking the alternatives.

Table 12 provides the ranking results of the various tourist destinations obtained using the three different MACBETH methods in different environments.

**Table 12:** Ranking results for each tourist destination using different methods.

Method	Ranking
MACBETH	$A_{1} \succ A_{3} \succ A_{2} \succ A_{4}$
IF-MACBETH	$A_{1} \succ A_{3} \succ A_{2} \succ A_{4}$
HFL-MACBETH	$A_{1} \succ A_{2} \succ A_{3} \succ A_{4}$
PL-MACBETH	$A_{1} \succ A_{3} \succ A_{2} \succ A_{4}$

It is evident that the four methods yield relatively consistent final ranking results, with destination *A*_1_ being the optimal choice. Notably, when the HFL-MACBETH method is introduced, the ranking sequence becomes $A_{1} \succ A_{2} \succ A_{3} \succ A_{4}$, which differs slightly from the other three methods (i.e., $A_{1} \succ A_{3} \succ A_{2} \succ A_{4}$). This indicates that although all methods maintain consistency in identifying the optimal alternative (A1), they differ in distinguishing the relative positions of the intermediate alternatives, reflecting variations in their capability to capture uncertainty and hesitation in linguistic expressions. This to a significant extent underscores the reliability and rationality of the methodology proposed in this study.

On the other hand, examining the specific final scores for each travel destination, under the classical MACBETH method, the destinations *A*_3_ and *A*_2_, ranked second and third, exhibit overall scores that are very close, differing by less than 1. Under the IF-MACBETH method, the gap between these two destinations widens to 4.2161, while under the PL-MACBETH method, the gap further expands to 8.3956, resulting in more distinct results. In contrast, the HFL-MACBETH method yields a relatively moderate separation between A2 and A3, falling between the IF-MACBETH and PL-MACBETH results, which suggests that it captures hesitation but does not fully represent probabilistic distribution of linguistic assessments.

This divergence can be attributed to the fact that the classical MACBETH method solely considers positive emotional information while neglecting neutral and negative emotions. In the IF-MACBETH method, although both positive and negative emotional information are accounted for, neutral emotional information is still overlooked. In contrast, the PL-MACBETH method takes into consideration all of the evaluation information mentioned above, with an emphasis on the probabilities of various evaluation information occurrences, minimizing the omission of original assessment data^31^. The HFL-MACBETH method, while capable of capturing hesitation in decision-makers’ linguistic judgments, primarily models uncertainty through possible linguistic term sets without explicitly incorporating probabilistic distribution information, which may limit its expressiveness in highly ambiguous online review environments.

Additionally, the utilization of PLTS to describe and characterize assessment information in the form of multi-valued weighted discrete linguistic term variables is not only more in line with the expressive manner of human thinking and perception but also offers greater flexibility and adaptability through the use of different granularity linguistic scales^34^. Moreover, the PL-MACBETH method is concise in approach, computationally efficient, and when integrated with techniques such as LDA and natural language processing, it becomes more practically significant^18^. Hence, the comparative results across the four methods (MACBETH, IF-MACBETH, HFL-MACBETH, and PL-MACBETH) further demonstrate that incorporating probabilistic linguistic information leads to more discriminative and stable ranking outcomes, and the PL-MACBETH method exhibits the strongest ability to differentiate closely competing alternatives while maintaining consistency in overall ranking structure.

#### Comparison with large language models (LLMs)

In the context of validating the proposed method’s effectiveness and reliability, it is crucial to compare its performance with that of large language models (LLMs) like ChatGPT. LLMs, known for their advanced semantic understanding and contextual processing capabilities, provide a robust benchmark for evaluating the comparative advantage of novel methods in data-driven research^35^. To this end, we utilize ChatGPT to conduct a semantic-based ranking of four alternative tourist destinations by inputting the raw textual review data directly into the model. ChatGPT ranks these tourist destinations by understanding their strengths and weaknesses across multiple dimensions, utilizing its semantic analysis and reasoning capabilities.

We further specify the experimental protocol for the LLM-based comparison to ensure reproducibility. The same input information (i.e., processed sentiment summaries and topic-level representations of the four destinations) was provided to ChatGPT (GPT-5, OpenAI) for ranking analysis. The following standardized prompt was adopted: “Based on the following structured information of four tourist destinations, please rank them from the most to least recommended in terms of overall tourist satisfaction and perceived destination image. Please provide a clear ranking and a brief justification”. To reduce randomness, the experiment was conducted three independent times using identical inputs and prompts. The outputs were highly consistent, and the majority voting strategy was used to determine the final LLM-based ranking result. The LLM input format was structured as summarized multi-dimensional sentiment-topic profiles rather than raw review texts to ensure fairness in comparison with the proposed method. Only the deterministic setting (temperature = 0) was used to minimize stochastic variation in generation. The results are reported in Table 13 (Ranking^1^). The output from ChatGPT produces a comprehensive ranking order of *A*_1_, *A*_3_, *A*_2_, and *A*_4_. Notably, this ranking is identical to that generated by the proposed method, thereby validating the accuracy and reliability of our approach. This congruence in results underscores the method’s capability to align with sophisticated language models in terms of understanding and processing complex, unstructured data.

**Table 13:** Ranking results for each tourist destination using ChatGPT.

	Dimension 1: Attraction appeal	Dimension 2: Tourist satisfaction	Dimension 3: Tour experience	Ranking^1^	Uncertainty level	Ranking^2^
*A*_1_	Very high, with significant historical and cultural value	High, but slightly lowered during holidays due to large crowds	Good, with convenient transportation and well-developed facilities	1	Medium	1
*A*_2_	High, with important historical significance	High, with beautiful surroundings	Good, but there are additional paid activities	3	Relatively low	2
*A*_3_	High, especially praised for its night views	High, with convenient transportation	Good, with favorable dining and accommodation	2	Relatively high	3
*A*_4_	High, with beautiful natural scenery	Average, with many additional paid activities	Average, with a tightly scheduled itinerary	4	Relatively high	4

ChatGPT considers the following dimensions. (1) Attraction appeal: Natural scenery, cultural landmarks, and historical and cultural value are included. (2) Tourist satisfaction: Service attitude, management quality, and cost-effectiveness are covered. (3) Tour experience: Ease of access, presence of additional paid activities, and availability of convenient services are included.

Furthermore, to assess the robustness of our method under conditions of uncertainty, we introduce noise into the data analysis. The inclusion of noise aims to simulate real-world data variability, thereby providing a more stringent test of the method’s stability. The final ranking order under these conditions is *A*_1_, *A*_2_, *A*_3_, and *A*_4_, as shown in Table 13 (Ranking^2^). The consistency of *A*_1_ remaining as the top-ranked destination and *A*_4_ as the lowest, despite the noise-induced shift between *A*_2_ and *A*_3_, strongly suggests that the proposed method maintains a high degree of robustness and reliability.

These findings are significant as they demonstrate not only the method’s effectiveness in a controlled setting but also its resilience when faced with data perturbations. The alignment with ChatGPT’s output and the method’s stability under noisy conditions collectively enhance the credibility and impact of the research, offering a compelling case for the method’s broader applicability in similar analytical contexts.

## Discussions

This study aims to address the challenges faced in the field of tourism, where travelers often struggle with intuitively perceived destination image and making informed tourist destination selection decisions. Through the integration of multiple methods, this study constructed a perceived destination image based on PLTS, successfully applying it to the evaluation of tourist destinations and introducing the PL-MACBETH method. The methodology and application presented in this research offer a novel approach to solving issues in the field of tourism. The introduction of PLTS, the fusion of various technologies, and the application of the PL-MACBETH method collectively form a comprehensive research framework, providing decision-makers and travelers in the tourism industry with more accurate and comprehensive information support.

To further clarify the positioning and originality of this study, it is necessary to systematically distinguish it from existing research that also integrates online review data with PLTS-based decision-making frameworks. Prior studies, such as Luo et al.^21,32,36^, have made important contributions to the application of PLTS in tourism evaluation. However, these studies primarily focus on attribute-level evaluation or static aggregation of linguistic assessments, rather than modeling the semantic and generative structure of tourists’ perceptions from unstructured textual data. From the perspective of data sources, existing studies typically rely on pre-constructed evaluation indicators or structured survey-based information, whereas this study directly utilizes large-scale online review data, which contain richer and more spontaneous expressions of tourist experiences. In terms of destination image construction logic, prior research often assumes predefined evaluation dimensions, while this study employs LDA-based topic modeling to endogenously extract multidimensional perception structures from text, thereby enabling a data-driven formation mechanism of destination image.

Regarding uncertainty representation, existing approaches based on classical fuzzy sets or linguistic term sets generally capture ambiguity in a deterministic or single-layer manner. In contrast, this study adopts PLTS to simultaneously represent both linguistic ambiguity and probability distribution, thereby providing a more expressive and fine-grained characterization of tourists’ subjective evaluations. For weight generation, previous studies mainly rely on expert judgment or direct aggregation mechanisms, whereas this study derives weights implicitly from topic-sentiment distributions extracted from user-generated content. In terms of decision and ranking methodology, earlier work often applies a single MCDM method for ranking purposes. Differently, this study integrates PL-MACBETH to handle multi-criteria evaluation under probabilistic linguistic environments, ensuring consistency between uncertainty representation and decision aggregation logic. Finally, with respect to application context, prior studies mainly focus on static evaluation of tourist destinations, while this research emphasizes an end-to-end analytical pipeline from unstructured review mining to perception modeling and decision-making, thereby enhancing both interpretability and practical applicability.

Overall, the theoretical contribution of this study lies in advancing destination image research from attribute-based static evaluation toward perception-driven semantic modeling. Methodologically, the novelty is reflected not in replacing existing ranking techniques, but in constructing an integrated framework that combines topic modeling, probabilistic linguistic uncertainty representation, and MCDM-based decision analysis to capture the complexity of tourist perception in online review environments.

### Contributions to theory

It is expected to have a positive impact on theoretical implications.

Firstly, the application of PLTS in this study offers significant advantages in dealing with the fuzziness and uncertainty inherent in evaluation information. Due to the flexibility and effectiveness of PLTS in representing online review information, it has garnered widespread recognition and acclaim from numerous scholars. Darko and Liang^37^ developed an improved probabilistic linguistic linear programming technique for consumer multidimensional preference analysis. Peng et al.^38^ introduced PLTS to aggregate a large volume of hotel-related review information from TripAdvisor for qualitative text processing. Cui et al.^14^ highlighted the significant uncertainty and fuzziness in consumer reviews, underscoring the utility of PLTS as a valuable tool. Yu et al.^39^ utilized PLTS to reveal rating levels and probability distributions in online hotel reviews. Zhang et al.^40^ proposed a new model based on probabilistic linguistic information processing to fully utilize and integrate ratings and reviews from multiple websites. Therefore, beyond existing applications that mainly treat PLTS as an aggregation or representation tool, this study further extends PLTS into a perception-level modeling mechanism that captures probabilistic distributions of tourists’ subjective expressions embedded in unstructured online review data. The introduction of PLTS in this study enables a more precise capture of complex emotions and attitudes contained in online traveler expressions, thus providing travelers with a more genuine reference and decision-makers with more specific data support. Accordingly, PLTS in this framework functions not only as an uncertainty modeling technique but also as a bridge connecting linguistic expression and structured perception representation.

Secondly, the techniques used in this study, such as the LDA topic extraction model and sentiment analysis, provide effective means for constructing a comprehensive and multidimensional perceived destination image based on online traveler expressions. By extracting topics from the expressions of travelers, various aspects of tourist destinations are revealed from multiple perspectives, enriching the content of the perceived destination image. The inclusion of sentiment analysis makes the construction of the perceived destination image emotionally resonant and more closely aligned with travelers’ real experiences and emotional needs. More importantly, rather than using LDA as a standalone text mining tool, this study embeds LDA within a perception construction framework, where latent topics are interpreted as structured dimensions of destination image. In particular, we propose a novel approach by integrating PLTS with LDA to more accurately represent the multidimensional features of tourist destinations in the context of destination image analysis. PLTS serves as a probabilistic linguistic model for handling qualitative text information, addressing ambiguity and uncertainty, while LDA acts as a topic model to uncover latent structures within the text^21,36^. Our integrated method leverages the flexibility of PLTS and the topic modeling capabilities of LDA to comprehensively analyze tourist destination evaluation data, extracting key thematic features relevant to destination image. Thus, this approach offers a fresh perspective and methodology for research on destination image. From a theoretical perspective, this integration establishes a structured pathway from unstructured textual expression to multidimensional perceptual representation, thereby enhancing the interpretability of destination image construction models.

Finally, the application of the PL-MACBETH method in this study provides efficient pre-trip decision support for travelers. This method extends the traditional MACBETH method into a probabilistic linguistic environment, taking better account of the fuzziness and uncertainty of evaluation information. This extension builds upon previous literature on decision support system and MCDM techniques, enhancing the applicability of the MACBETH method in the context of probabilistic linguistic modeling^41^. More specifically, this study contributes to the literature by extending MACBETH from a deterministic preference modeling framework to a probabilistic linguistic decision-making environment, where uncertainty is explicitly embedded into preference aggregation. By incorporating probabilistic linguistic structures, the PL-MACBETH method offers a more nuanced framework for travelers to make informed decisions in the face of uncertain and ambiguous evaluation data. Besides, the integration of the PL-entropy weight method enhances the specificity and scientific nature of ranking recommendations. Hence, this method is expected to offer travelers wiser choices, ultimately enhancing the quality of the travel experience. Overall, this study constructs an integrated theoretical pipeline that connects semantic perception modeling, probabilistic uncertainty representation, and multi-criteria decision analysis in a unified framework for tourism evaluation.

### Implications to practice

The methodology and application of this study hold significant practical implications in addressing challenges within the tourism industry, positively impacting the competitiveness of tourist destinations and the quality of the traveler experience. The following three practical insights are derived from this study.

Firstly, the study emphasizes the need for strengthening the management of perceived destination image. The constructed perceived destination image based on PLTS, with its multidimensional and finely-grained depiction, accurately presents the true condition of destinations. This provides powerful tools for destination managers to better shape and manage their image. Destination managers can leverage the fuzzy expressions of travelers to understand their impressions and expectations, and consequently, tailor their marketing, promotion, and service improvements to enhance the attractiveness and reputation of the destination.

Secondly, there is an opportunity to enhance the scientific nature and personalization of travel decisions. The PL-MACBETH method proposed in this study provides efficient pre-trip decision support for travelers. This method, by considering the fuzziness and uncertainty of online traveler expressions, offers more precise ranking recommendations for tourist destinations. In practical applications, travelers can choose destinations that better suit their personalized preferences based on their interests and needs. This contributes to improved decision-making and satisfaction in travel choices.

Lastly, the use of sentiment analysis techniques in this study makes the construction of perceived destination image emotionally resonant. Travelers often consider emotional experiences when choosing a tourist destination, alongside practical information. Therefore, tourist destinations can use sentiment analysis results to better capture and meet the emotional needs of travelers. Strengthening the communication of emotional resonance in service design and marketing promotions is likely to deliver more profound and memorable travel experiences.

## Conclusions

### Summary

To address the challenges of travelers not being able to intuitively perceive the destination image and make tourist destination selection decisions, this study leverages fuzzy expressions from online travelers. It integrates LDA topic extraction models, sentiment analysis, and PLTS theory to construct a perceived destination image based on PLTS. This approach provides a multidimensional and finely-grained representation of the real conditions of the tourist destination. Building upon this foundation, the study extends the classical MACBETH method into a probabilistic linguistic environment, presenting the PL-MACBETH method. The method is successfully applied to the evaluation of tourist destinations, offering efficient pre-trip decision support to travelers. The comparative analysis underscores the effectiveness and superiority of this approach from various perspectives.

In conclusion, based on the research findings, the following points are established.

First, PLTS is capable of better capturing fuzziness and uncertainty in handling evaluation information, leading to a more accurate depiction of the perceived destination image.

Second, by utilizing techniques such as LDA topic extraction and sentiment analysis on online traveler expressions, a more comprehensive and multidimensional perceived destination image is constructed, providing more valuable information for travelers’ decision-making.

Finally, the application of the PL-MACBETH method, in conjunction with the PL-entropy weight method, effectively ranks and recommends tourist destinations, offering more tailored recommendations to travelers.

### Limitation and future research

This study not only provides innovative solutions to problems in the tourism field at the theoretical level but also offers valuable insights for practical applications related to destination management, travel decision-making, and enhancing the travel experience. With ongoing technological advancements and the wider adoption of these insights, they are expected to contribute to the sustainable development of the tourism industry and increased traveler satisfaction. However, this study still has certain limitations.

On the one hand, as traveler fuzzy expression information becomes increasingly complex and diverse in terms of information types and structures, PLTS effectively represents and characterizes this information^42^. The expansion of their applications and their organic integration with other theories or technologies to serve travelers, travel destinations, and the entire tourism industry remains a promising area for future research.

On the other hand, we suggest integrating visual content analysis with textual analysis to comprehensively understand destination images. Textual analysis provides descriptions and contexts of destinations, while visual content analysis captures visual features and emotional information from images^43^. This integration may facilitate a deeper exploration of the significant role visual content plays in shaping destination perceptions. Future research could explore this integrated approach and investigate its potential value in revealing destination images. Such research endeavors would offer richer insights and guidance for both the tourism industry and academia, thereby advancing the study of destination imagery in tourism.

## Data Availability

The datasets used and/or analyzed during the current study available from the corresponding author on reasonable request.

## References

[1] Sharma, P. Destination evangelism and engagement: Investigation from social media-based travel community. *Electron. Commer. Res. Appl.* **57**, 101228. 10.1016/j.elerap.2022.101228 (2023).

[2] Lv, X., Zhang, C. & Li, C. Beyond image attributes: A new approach to destination positioning based on sensory preference. *Tour. Manag.* **100**, 104819. 10.1016/j.tourman.2023.104819 (2024).

[3] Molinillo, S., Japutra, A. & Ekinci, Y. Building brand credibility: The role of involvement, identification, reputation and attachment. *J. Retail. Consum. Serv.* **64**, 102819. 10.1016/j.jretconser.2021.102819 (2022).

[4] Wang, X. et al. How to perceive tourism destination image? A visual content analysis based on inbound tourists’ photos. *J. Destin. Mark. Manag.* **33**, 100923. 10.1016/j.jdmm.2024.100923 (2024).

[5] Wang, X., Zhang, C. & Xu, Z. A product recommendation model based on online reviews: Improving PageRank algorithm considering attribute weights. *J. Retail. Consum. Serv.* **81**, 104052. 10.1016/j.jretconser.2024.104052 (2024).

[6] Huang, Y., Wu, W. & Qian, L. Explicit vs. implicit? How different self-presentations of luxury tourism experience trigger others’ value co-destruction intention in online travel communities. *Tour. Manag.* **107**, 105058. 10.1016/j.tourman.2024.105058 (2025).

[7] Liu, Y., Zhang, X., Zhang, H. & Zha, X. Competing tourism service provider introduction strategy for an online travel platform with demand information sharing. *Electron. Commer. Res. Appl.* **49**, 101084. 10.1016/j.elerap.2021.101084 (2021).

[8] Qin, Y., Luo, C., Luo, Y. & Ngai, E. W. Configurational patterns for forecasting customer satisfaction enhancement based on online reviews: A multi-attribute attitude perspective. *Inf. Process. Manag.* **63**(3), 104545. 10.1016/j.ipm.2025.104545 (2026).

[9] Lalicic, L., Marine-Roig, E., Ferrer-Rosell, B. & Martin-Fuentes, E. Destination image analytics for tourism design: An approach through Airbnb reviews. *Ann. Tour. Res.* **86**, 103100. 10.1016/j.annals.2020.103100 (2021).

[10] Ray, R. K. & Singh, A. From online reviews to smartwatch recommendation: An integrated aspect-based sentiment analysis framework. *J. Retail. Consum. Serv.* **82**, 104059. 10.1016/j.jretconser.2024.104059 (2025).

[11] Wang, H., Jiang, G., Hong, M. & Abdalbari, H. Graph-based bootstrapped latent recommendation model. *Electron. Commer. Res. Appl.* **68**, 101446. 10.1016/j.elerap.2024.101446 (2024).

[12] Wu, W. & Law, R. Evaluation of generative artificial intelligence tools in tourism using the extended VIKOR method with probabilistic linguistic information incorporating regret theory. *Inf. Technol. Tour.* **28**(1), 10. 10.1007/s40558-025-00341-3 (2026).

[13] Qin, Y., Luo, C. & Ngai, E. W. Deconstructing customer satisfaction recipes: A dynamic configurational framework leveraging the power of online reviews in tourism contexts. *Tour. Manag.* **110**, 105181. 10.1016/j.tourman.2025.105181 (2025).

[14] Cui, C., Wei, M., Che, L., Wu, S. & Wang, E. Hotel recommendation algorithms based on online reviews and probabilistic linguistic term sets. *Expert Syst. Appl.* **210**, 118503. 10.1016/j.eswa.2022.118503 (2022).

[15] Qin, Y., Wang, X. & Xu, Z. Ranking tourist attractions through online reviews: A novel method with intuitionistic and hesitant fuzzy information based on sentiment analysis. *Int. J. Fuzzy Syst.* **24**(2), 755–777. 10.1007/s40815-021-01131-9 (2022).

[16] Zadeh, L. A. Fuzzy sets. *Inf. Control* **8**(3), 338–353. 10.1016/S0019-9958(65)90241-X (1965).

[17] Ranjbar Kermany, N. & Alizadeh, S. H. A hybrid multi-criteria recommender system using ontology and neuro-fuzzy techniques. *Electron. Commer. Res. Appl.* **21**, 50–64. 10.1016/j.elerap.2016.12.005 (2017).

[18] Wu, X., Liao, H. & Tang, M. Product ranking through fusing the wisdom of consumers extracted from online reviews on multiple platforms.. *Knowl.-Based Syst.* **284**, 111275. 10.1016/j.knosys.2023.111275 (2024).

[19] Wang, L., Wang, X.-K., Peng, J.-J. & Wang, J.-Q. The differences in hotel selection among various types of travellers: A comparative analysis with a useful bounded rationality behavioural decision support model. *Tour. Manag.* **76**, 103961. 10.1016/j.tourman.2019.103961 (2020).

[20] Pang, Q., Wang, H. & Xu, Z. Probabilistic linguistic term sets in multi-attribute group decision making. *Inf. Sci.* **369**, 128–143. 10.1016/j.ins.2016.06.021 (2016).

[21] Luo, Y., Zhang, X., Qin, Y., Yang, Z. & Liang, Y. Tourism attraction selection with sentiment analysis of online reviews based on probabilistic linguistic term sets and the idocriw-cocoso model. *Int. J. Fuzzy Syst.* **23**(1), 295–308. 10.1007/s40815-020-00969-9 (2021).

[22] Wu, X., Liao, H., Xu, Z., Hafezalkotob, A. & Herrera, F. Probabilistic linguistic MULTIMOORA: A multicriteria decision making method based on the probabilistic linguistic expectation function and the improved Borda rule. *IEEE Trans. Fuzzy Syst.* **26**(6), 3688–3702. 10.1109/TFUZZ.2018.2843330 (2018).

[23] Bana e Costa, C. A. & Chagas, M. P. A career choice problem: An example of how to use Macbeth to build a quantitative value model based on qualitative value judgments. *Eur. J. Oper. Res.* **153**(2), 323–331. 10.1016/S0377-2217(03)00155-3 (2004).

[24] Gkouvitsos, I. & Giannikos, I. Using a MACBETH based multicriteria approach for virtual weight restrictions in each stage of a DEA multi-stage ranking process. *Oper. Res.* **22**(3), 1787–1811. 10.1007/s12351-020-00619-w (2022).

[25] Shashank, S. & Behera, R. K. Factors influencing recommendations for women’s clothing satisfaction: A latent Dirichlet allocation approach using online reviews.. *J. Retail. Consum. Serv.* **81**, 104011. 10.1016/j.jretconser.2024.104011 (2024).

[26] Zhao, X. & Huang, Z. A method for exploring consumer satisfaction factors using online reviews: A study on anti-cold drugs. *J. Retail. Consum. Serv.* **81**, 103895. 10.1016/j.jretconser.2024.103895 (2024).

[27] Zheng, L., Sun, L., He, Z. & He, S. Dynamic product competitive analysis based on online reviews. *Decis. Support Syst.* **183**, 114268. 10.1016/j.dss.2024.114268 (2024).

[28] Blei, D. M., Ng, A. Y. & Jordan, M. I. Latent Dirichlet allocation. *J. Mach. Learn. Res.* **3**(Jan), 993–1022 (2003).

[29] Bani-Doumi, M., Serrano-Guerrero, J., Chiclana, F., Romero, F. P. & Olivas, J. A. A picture fuzzy set multi criteria decision-making approach to customize hospital recommendations based on patient feedback. *Appl. Soft Comput.* **153**, 111331. 10.1016/j.asoc.2024.111331 (2024).

[30] Calderón-Fajardo, V., Anaya-Sánchez, R. & Molinillo, S. Understanding destination brand experience through data mining and machine learning. *J. Destin. Mark. Manag.* **31**, 100862. 10.1016/j.jdmm.2024.100862 (2024).

[31] Liu, F., Liao, H. & Al-Barakati, A. Physician selection based on user-generated content considering interactive criteria and risk preferences of patients. *Omega (Oxf.)* **115**, 102784. 10.1016/j.omega.2022.102784 (2023).

[32] Luo, Y., He, J., Yang, Z., Wang, J. & Li, R. Exploring the destination image based on the perspective of tourists’ expression using machine learning methods combined with plts-pt. *Soft Comput.* **27**(9), 5537–5552. 10.1007/s00500-023-07815-8 (2023).

[33] Zheng, W.-Q., Cheung, S.-M., Zhu, B.-W., Xiong, L. & Tzeng, G.-H. A hybrid multi-attribute decision-making model for the systematic evaluation of exoticism-themed retail spaces from the perspective of consumer experience. *J. Retail. Consum. Serv.* **79**, 103848. 10.1016/j.jretconser.2024.103848 (2024).

[34] Zhang, C., Xu, Z., Gou, X. & Chen, S. An online reviews-driven method for the prioritization of improvements in hotel services. *Tour. Manag.* **87**, 104382. 10.1016/j.tourman.2021.104382 (2021).

[35] Yi, J., Oh, Y. K. & Kim, J.-M. Unveiling the drivers of satisfaction in mobile trading: Contextual mining of retail investor experience through BERTopic and generative AI. *J. Retail. Consum. Serv.* **82**, 104066. 10.1016/j.jretconser.2024.104066 (2025).

[36] Luo, Y., He, J., Mou, Y., Wang, J. & Liu, T. Exploring China’s 5A global geoparks through online tourism reviews: A mining model based on machine learning approach. *Tour. Manag. Perspect.* **37**, 100769. 10.1016/j.tmp.2020.100769 (2021).

[37] Darko, A. P. & Liang, D. A heterogeneous opinion-driven decision-support model for tourists’ selection with different travel needs in online reviews. *J. Oper. Res. Soc.* **74**(1), 272–289. 10.1080/01605682.2022.2035274 (2023).

[38] Peng, H.-G., Zhang, H.-Y. & Wang, J.-Q. Cloud decision support model for selecting hotels on TripAdvisor.com with probabilistic linguistic information. *Int. J. Hosp. Manag.* **68**, 124–138. 10.1016/j.ijhm.2017.10.001 (2018).

[39] Yu, S.-m, Du, Z.-j, Lin, X.-d, Luo, H.-y & Wang, J.-q. A stochastic dominance-based approach for hotel selection under probabilistic linguistic environment. *Mathematics* **8**(9), 1525 (2020).

[40] Zhang, Y., Liang, D. & Xu, Z. Cross-platform hotel evaluation by aggregating multi-website consumer reviews with probabilistic linguistic term set and Choquet integral. *Ann. Oper. Res.*10.1007/s10479-022-05075-7 (2022). 36415819 10.1007/s10479-022-05075-7 PMC9672591

[41] Pu, Z., Zhang, C., Xu, Z. & Wang, X. A fuzzy decision support model for online review-driven hotel selection by considering risk attitudes of customers. *J. Oper. Res. Soc.* **75**(7), 1407–1420. 10.1080/01605682.2023.2249938 (2024).

[42] Krishankumar, R. et al. An integrated personalized decision approach with probabilistic linguistic context for grading restaurants in India. *Appl. Soft Comput.* **136**, 110089. 10.1016/j.asoc.2023.110089 (2023).

[43] Qian, L., Guo, J., Qiu, H., Zheng, C. & Ren, L. Exploring destination image of dark tourism via analyzing user generated photos: A deep learning approach. *Tour. Manag. Perspect.* **48**, 101147. 10.1016/j.tmp.2023.101147 (2023).

